# Transcatheter ablation of atrioventricular nodal reentry tachycardia in children and congenital heart disease in the era of 3D mapping

**DOI:** 10.3389/fcvm.2024.1506858

**Published:** 2024-11-28

**Authors:** Fabrizio Drago, Francesco Flore, Cristina Raimondo, Claudio Pandozi

**Affiliations:** Paediatric Cardiology and Cardiac Arrhythmias Complex Unit, Bambino Gesù Children’s Hospital IRCCS, Rome, Italy

**Keywords:** atrioventricular nodal reentry tachycardia, 3D mapping, transcatheter ablation, children, congenital heart disease

## Abstract

Atrioventricular nodal reentrant tachycardia (AVNRT) is a common supraventricular tachycardia in children and congenital heart disease (CHD) patients. Nowadays, in large enough children, chronic treatment for symptomatic and recurrent AVNRT episodes relies on transcatheter ablation. Indeed, many three-dimensional (3D) mapping strategies and ablation techniques have been developed and it helped to increase success rates and to reduce complications. Therefore, this study aimed to perform an updated comprehensive review of the available literature regarding contemporary management of AVNRT in children. A literature search was performed using Google Scholar, PubMed, Springer, Ovid, and Science Direct. We found that in recent times many investigations have demonstrated that 3D mapping systems allow to localize more precisely the ablation substrate, with minimal use of fluoroscopy. The most frequently employed mapping strategies are the low-voltage bridge strategy together with the search for the SP potential and the Sinus Rhythm Propagation Map with the identification of areas of Wave Collision or Pivot Points. For transcatheter ablation in pediatric settings, radiofrequency (RF) ablation was first used in the 1990s, while cryoablation was introduced in 2003 and nowadays represents the most used energy for AVNRT ablation in this population. Indeed, its specific features, such as reversible cryomapping, cryoadhesion and the precision in lesion delivery, made this technique very appealing to decrease complications and fluoroscopy time. As regards AVNRT in CHD patients, it represents the third most common form of arrhythmia in children with CHD. However, in this subgroup ablation remains challenging and experience limited, since anatomy may be atypical and the areas of ablation less predictable or less accessible.

## 1 Introduction

Atrioventricular nodal reentrant tachycardia (AVNRT) is a common, regular, narrow complex QRS tachycardia in children, accounting for 13%–34% of supraventricular tachycardias (SVT) in this population (1–4). Its incidence increases with age, being extremely rare in infants younger than 2 years of age (1%–10% of SVT) and almost as frequent as atrioventricular reentry tachycardia (AVRT) in females older than 12 years (up to 44% of SVT) (4–6). Pediatric patients present with various and often atypical symptoms, and the diagnosis is achieved through ECG and electrophysiology study (EPS) (7).

As for AVNRT in adults, the most common type of AVNRT uses the so-called slow nodal pathway (SP) as the anterograde limb of the reentrant circuit and the so-called fast pathway (FP) as the retrograde limb (“slow-fast” or typical AVNRT). More rarely, the reentry circuit is reversed (“fast-slow” AVNRT) or uses nodal pathways with “intermediate” electrophysiological properties; all these tachycardias are referred to as atypical AVNRT (7–11).

In recent years, three-dimensional (3D) mapping systems allowed to localize more precisely the SP, with minimal use of fluoroscopy. In addition, new ablation strategies focused on improving success rates and reducing complications. In this article, we provide an updated summary of AVNRT mapping and ablation in pediatric and CHD patients based on an extensive review of the literature.

## 2 Anatomy and histology of the nodal conduction system

In the era of 3D mapping and transcatheter (TC) ablation, appropriate knowledge of the atrioventricular node (AVN) anatomy has become imperative for appropriate invasive treatment of AVNRT.

**Figure 1:**
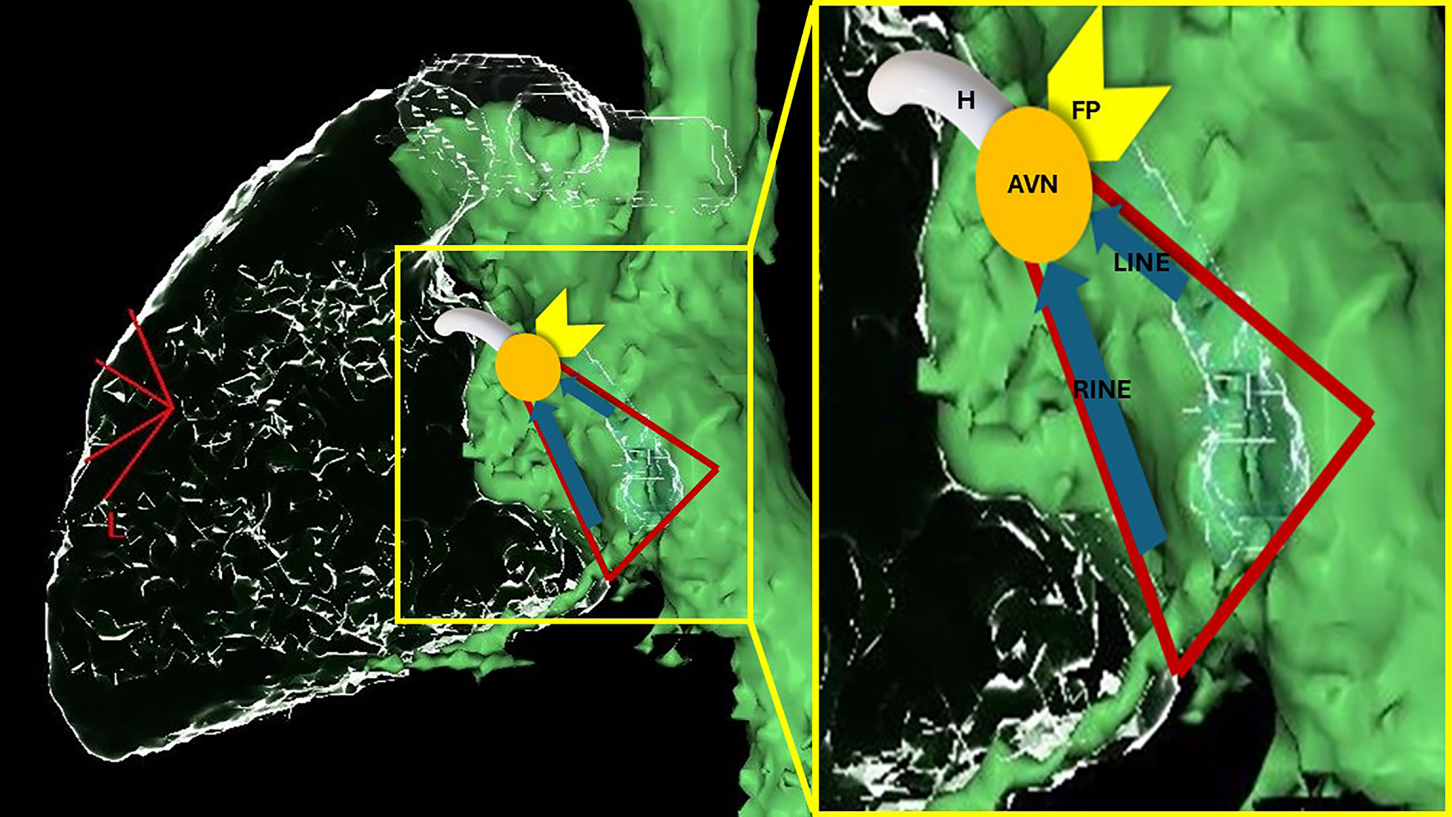
Schematic representation of AV nodal structures superimposed on a computed-tomography reconstruction of the right cardiac chambers from a left lateral view (the green chamber is a reconstruction of right atrium, inferior and superior vena cava; the glass structures represent right ventricle, pulmonary artery and coronary sinus). The triangle of Koch is marked in red. RINE, right inferior nodal extension; LINE, left inferior nodal extension; AVN, atrioventricular node; FP, fast pathway; H, His.

Morphologically, the AVN can be subdivided into three zones: the lower nodal bundle (LNB), the compact node (CN), and the inferior extensions (INE), namely the right inferior nodal extension (RINE) and the left inferior nodal extension (LINE) (Figure 1). Moreover, in the area between the AVN and the adjacent working myocardium, special cells called “transitional cells” surround the proximal margins of the CN and, more extensively, the inferior extensions towards the ostium of the coronary sinus and Eustachian ridge. These cells exhibit an intermediate morphology between the cells of the CN and those of the working myocardium (12–16).

Interestingly, the composition of the nodal structures has been reported to change with growth, such that the modifications already are notable in pediatric patients. Indeed, fibrofatty tissue gradually increases in the CN, creating a looser texture, and the gradual expansion of the muscular AV septum determines a CN more or less “glued” to the right crest of the septum with a more fusiform shape. Moreover, the RINE and LINE become significantly longer in young adults than in infants; therefore, their position changes from relatively close together in very young children to quite distant in adolescents and young adults. In addition, the intensity of contacts between the transitional cells and the AVN diminishes considerably, especially in the anterior parts. This, together with the modifications in length and relationships of the INE, may “set the scene” for AVNRT and could explain why this condition is more frequent in young adults than in infants. Indeed, it is thought that with increasing age, the non-uniform, anisotropic activation in the Koch's triangle (KT) increases, thus enhancing the probability of reentry in case of appropriately timed extrasystoles (13–15, 17).

After puberty and especially in adulthood, the LNB occupies the anterior portion of the AVN and is composed of large cells oriented parallel to each other, whilst the CN is an oval structure composed of smaller, densely packed, irregularly shaped cells located at the apex of KT, with distinct cellular histology, and fewer intercellular connections. As regards its INE, the RINE runs close to the annular attachment of the septal tricuspid valve leaflet, extends to the level of the ostium of the coronary sinus, and is connected anteriorly with both the compact AVN and the LNB. The LINE is positioned superiorly to the RINE at the level of the roof of the CS and diverts leftward toward the central fibrous body and the mitral annulus. The LINE is usually much shorter than the RINE, and the 2 extensions merge proximally to form the base of the compact AVN. Of note, in some individuals, the LINE is not present (14, 15, 17–19).

According to experimental, electrophysiology and anatomical studies, the INE, particularly the RINE and its surrounding transitional cells, have been perceived as the substrate of the slow pathway (SP) in humans, as discussed in the following chapter (12, 17, 19).

In the approach of pediatric patients, it has also to be highlighted that the length of KT is correlated with age, body surface area and the dimensions of the tricuspid valve (while in adults it poorly correlates with these parameters). For example, KT has been reported to be, on average, 7.8 mm in patients less than one year of age, 11.1–15.6 mm for BSA 0.8 m2, 17.9 mm in adolescents aged >12 (13, 20, 21).

Congenital heart disease (CHD) patients may present with AVNRT at any age and more frequently when pressure or volume overload of the right heart exists, probably because of cellular electrophysiological alterations and intercellular fibrosis with consequent anisotropic conduction in the nodal/peri-nodal tissue (22, 23). In addition, in children affected by CHD, the AVN location is less predictable, and landmarks for KT can be distorted in some conditions (24).

## 3 Clinical presentation and ECG features

Pediatric patients suffering from AVNRT may present with palpitations, either at rest or with faster than normal heart rate during exercise, dizziness, syncope, chest pain, shortness of breath, sensation of pulsations in the throat. Infants and non-verbal toddlers may exhibit signs of poor feeding, profuse sweating with feeding, fast breathing and/or they can appear generally ill. Signs/symptoms usually have sudden onset and termination and can be brief but can also last for several hours (7, 9, 10).

**Figure 2:**
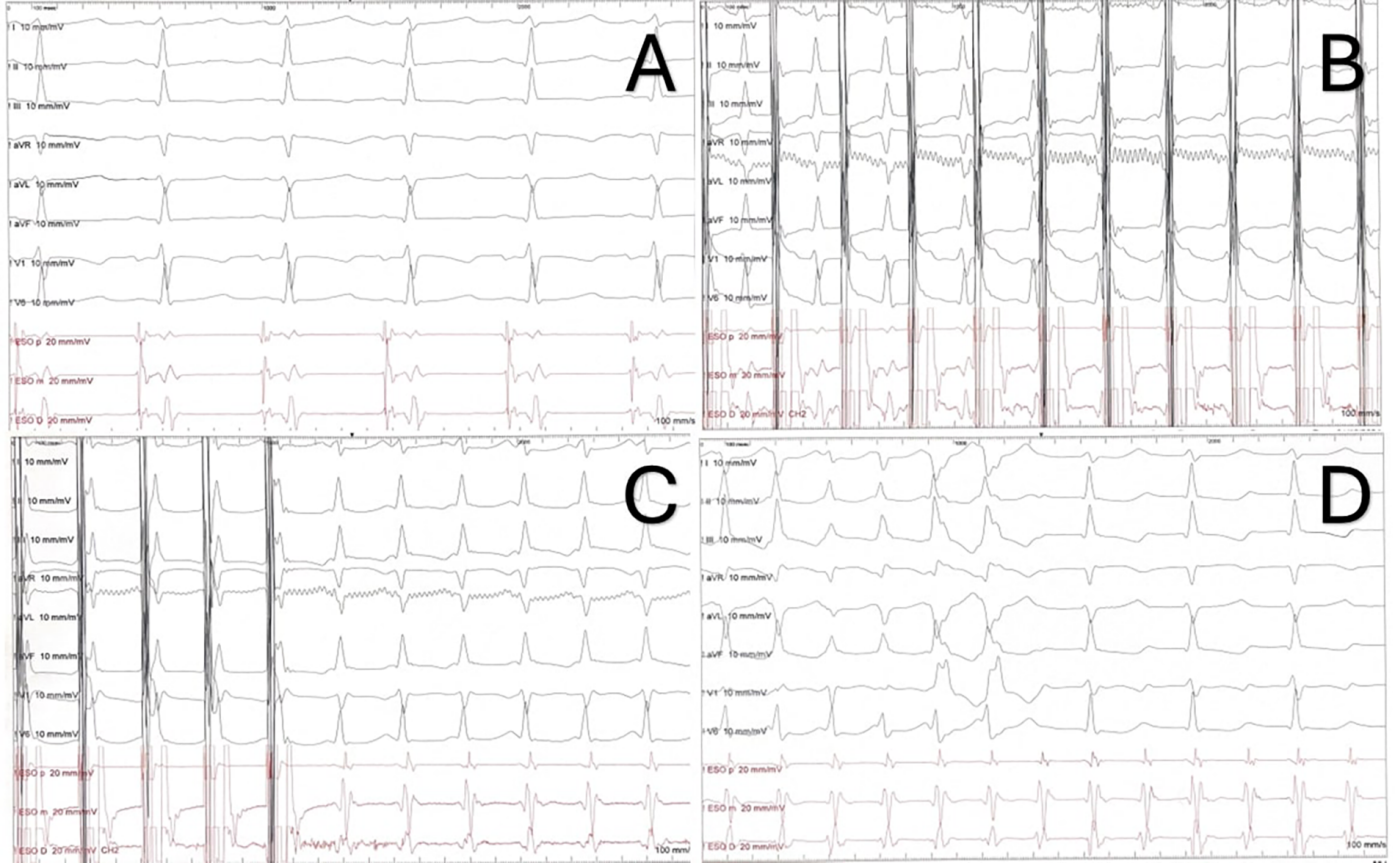
ECG and electrophysiology properties of AVNRT. **(A)** ECG and transesophageal electrograms in sinus rhythm. **(B)** Decremental atrial stimulation showing a Kay sign. **(C)** Induction of typical AVNRT through decremental atrial stimulation. Note the short RP (and VA) with pseudo-r’ in lead V1. **(D)** Same patient from the recordings in panel (A–C). 1:1 AV conduction in the first part of the strip, followed by intraventricular conduction with right bundle branch block (two beats) and 2:1 AV conduction.

Baseline ECG is usually normal (Figure 2A). During tachycardia, most often it shows a regular, narrow complex QRS tachycardia; heart rate may be higher than 250 bpm, especially in younger children, but the heart rate strongly depends on the adrenergic tone (9, 10, 25). Rarely, the tachycardia may present with functional bundle branch block (as a wide QRS complex tachycardia) or AV conduction may be blocked in a 2:1 ratio (7, 11, 26) (Figure 2D).

In AVNRT, typically the retrograde P waves are hidden in the terminal portion of the QRS. However, in the typical form, sometimes they create a pseudo-r’ in lead V1 or a pseudo-s wave in the inferior leads. In these cases, the RP interval is very short, less than 70 ms; therefore, typical AVNRT is classified as a short-RP tachycardia. In atypical forms, the P waves are visible at the end of or just after the T waves (long RP tachycardia) and have a negative polarity in the inferior leads, thus mimicking atrial tachycardias arising in the posteroseptal region or AVRT mediated by posteroseptal accessory pathways (8, 10, 11, 25).

There are no specific triggers for AVNRT; nonetheless, it is often associated with increased adrenergic tone. It is common for children to have their first (and subsequent) episodes of AVNRT during sporting events. Unfortunately, once a patient has been diagnosed with AVNRT there is a high recurrence rate, affecting the quality of life. On the other hand, episodes of AVNRT are usually self-limiting and do not result in tachycardia-induced cardiomyopathy (8–10, 25).

## 4 Electrophysiological properties

Several forms of AVNRT and possible circuits have been described. However, mapping and ablation strategies in children largely overlap and may differ slightly only between typical and atypical AVNRT (VA <60–70 ms and VA >60–70 ms, respectively) (27, 28).

The diagnosis of AVNRT in the electrophysiology (EP) lab may be slightly less obvious in children than in adults. Indeed, it usually relies on:

- The evidence of dual AVN physiology. During programmed atrial stimulation, the ﬁnding of an “AH jump”, that is a ≥50 ms increase in A2-H2 time with a 10 ms decrement in A1-A2 paced interval. During decremental atrial stimulation, a PR interval greater than or equal to RR (“Kay sign”) (Figure 2B).
- SVT features: VA relationship with V = A, VA <70 ms, concentric atrial activation (in typical forms).
- Responses to pacing maneuvers: “AH response” to ventricular overdrive pacing; a post-pacing interval minus tachycardia cycle length (PPI−TCL) following entrainment from the RV apex of more than 115 ms; an absolute “preexcitation index” greater than 100 ms (11, 27, 29). Importantly, no single pacing maneuver is 100% sensitive/specific for certain SVT (for instance, false positive ventricular pacing maneuver can be seen with a bystander, concealed nodo-fascicular/nodo-ventricular accessory pathway in a case of AVNRT) (30); however, a more detailed description of diagnostic pacing maneuvers goes beyond the purpose of this review and is presented elsewhere.

However, in the pediatric setting, the clinical tachycardia may not be inducible during the EP study, because general anesthesia is usually required (especially in younger children) and it diminishes overall tachyarrhythmias inducibility (31). Moreover, the AH jump and the Kay sign are less common than in adults (26, 27, 32–34). In detail, an AH jump is reported to be present in 42%–64% of pediatric AVNRT patients vs. 30% of pediatric patients without AVNRT; the maximum AH achieved is overall longer but with a large overlap between AVNRT and non-AVNRT children; a Kay sign is present in almost 2/3 of AVNRT patients vs. a minority of controls (26, 33, 35, 36). In fact, due to a more rapid nodal conduction in children, a 35–40 ms jump or a discontinuity in A2-H2 time is considered enough by some EP labs to establish the diagnosis of dual AVN physiology (25).

Additionally, in children a minimally invasive EP study can be obtained by transesophageal atrial pacing (TAP, Figure 2). Over the years, it has proven to be a highly accurate in diagnosing and characterizing various SVT in pediatric patients (37–40). Indeed, through atrial stimulation it is possible to study the AV conduction and to induce SVTs. Furthermore, TAP can be helpful in differential diagnosis of these tachycardias: in typical AVNRT, the VA interval <60–70 ms, with a P wave in lead V1 either not discernible or negative/biphasic and simultaneous with the atrial EGM (Figure 2C); in atypical AVNRT the VA interval >60–70 ms, the P wave is positive in D1 and the P wave in V1 occurs before esophageal atrial EGM (28, 41, 42).

## 5 Mapping strategies

In the recent years, mapping strategies for AVNRT in pediatric patients have evolved to a fluoroless approach using 3D mapping systems (43–45). Even though only one recent trial (46) focused on direct comparison of 3D mapping vs. conventional fluoroscopic approach in children, data suggest that safety/efficacy of AVNRT ablation have improved in the 3D mapping era. Indeed, with 3D mapping systems almost all studies report 100% acute success rate and very low recurrences; moreover, permanent AV block was never described, even when RF was used (see Table 1). On the other hand, a meta-analysis on studies published before 2014 revealed that the mean acute procedural failure rate was 3.1% for cryoablation and 2.2% for RF ablation, and the mid-term failure (>2 months) was 9.7% for cryoablation and 3.8% for RF ablation; in addition, permanent AV block incidence was 0.75% for fluoroscopic RF ablation (57).

**Table 1:** Outcomes of transcatheter ablation in the era of 3D mapping systems.

Author	Journal	Year	Patients number	Age	Mapping strategy	Ablation energy	Acute success	Recurrence
Malloy (47)	Pediatr Cardiol	2014	28	15	LVB	CRYO	100%	14.2%
Bearl (48)	C Heart Dis.	2015	28	15.3	LVB	CRYO (first) + RF	96%	3.5%
Reddy (46)	HR	2017	71	14.3	LVB	CRYO	98.5%	0
Drago (49)	Europace	2018	35	12.1	LVB + SP potential	CRYO	100%	0
Drago (50)	Europace	2020	184	13	LVB + SP potential	CRYO	99.2%	2.7%
Drago (51)	JICE	2021	21	13	LVB (HD grid)	CRYO	100%	0
Drago (43)	PACE	2023	14	6.5	LVB + SP potential	CRYO	100%	0
Van Aartsen (52)	JICRMM	2017	39	15	Wave collision	CRYO	100%	2.8%
Fogarty (53)	JICRMM	2023	52	15	Wave collision	CRYO	-	-
Tseng (54)	HR	2023	36	16.5	Wave collision + LVB	CRYO	100%	5.6%
O'Leary (55)	JICE	2023	13	15.7	OT pivot point LVB Peak f>340 Hz	Random RF + CRYO	100%	7.6%
Howard (56)	JCE	2022	46	14	RIPPLE MAP	CRYO	100%	0
Total							99.2%	3.3%

All strategies described in children rely on electro-anatomical maps of the KT area during sinus activation and the direct recording of SP potentials (43, 46, 49–58).

Indeed, for both typical and atypical AVNRT, the ablation target is the SP. In this regard, Jackman and Haissaguerre first described the characteristics of atrial electrogram of SP in patients with AVNRT (59, 60). The “Jackman potential” (JP) (59, 61) is characterized by a low-frequency, low-amplitude component followed by a large-amplitude, high-frequency component (“hump and spike” pattern). The “Haissaguerre potential” (HP) (60) is a slow, hump, rounded potentials at the mid or posterior septum, anterior to the coronary sinus ostium, usually at two-thirds anterior-one-third posterior of the area between the His bundle and the coronary sinus ostium. This potential is more anterior in the right atrial septum compared to the JP, where the A:V ratio is approximately 0.7–0.8.

In his work, Jackman et al. explained the conduction properties of the SP in patients who had retrograde conduction over this pathway. Selective retrograde conduction over the SP was obtained by inducing either an atypical AVNRT or single echo beats that used the SP for retrograde conduction or by techniques of ventricular pacing that resulted in retrograde block in the FP. The earliest atrial activation during such conduction was recorded and located by mapping the right atrial septum and the coronary sinus. The sites of earliest atrial activation during retrograde SP conduction were located in the posterior septum, near the coronary sinus ostium. As mentioned, EGMs at these sites in SR showed a small atrial potential with the “hump and spike” feature and a large ventricular potential. The high-frequency atrial component generally has a late timing during sinus rhythm, while it represents the first retrograde atrial potential during fast-slow AVNRT. This potential was used to identify the presumed atrial insertion of the SP, and thus, as an ablation target in Jackman's study.

The authors also suggested the presence of either multiple atrial insertions of the SP or multiple SPs in some patients since they sometimes observed that conduction over the SP was affected by energy delivered at two to four different sites.

In contrast to the SP location, they also provided evidence that the atrial insertion of the FP is usually located in the anterior septum, close to the His bundle.

Similarly, Haissaguerre et al. attempted to map the FP and the SP in patients with AVNRT. They recorded slow, hump, rounded potentials at the mid or posterior septum, anterior to the coronary sinus ostium, usually at two-thirds anterior-one-third posterior of the area between the His bundle and the coronary sinus ostium. In addition, they described that these potentials show a typical response to increasing atrial rates: a strong decline in amplitude and slope, an increase in duration, and a separation from preceding atrial potentials until the disappearance of the EGM.

Haisaguerre et al. usually performed ablation at a site where these hump-like potentials were preceded by spiky atrial EGM, which is more anterior in the right atrial septum compared to the JP, where the A:V ratio was approximately 0.7–0.8.

In the following years, hybrid EGMs with characteristics of both these signals, recorded in slightly different locations within the KT, were reported, mainly in adults (61, 62). However, many studies in the following years targeted the SP potential in the mid/posterior part of the KT, where a JP could be registered (44, 49, 51, 63–65).

Other studies, using optical mapping, showed that the presence of two distinct components of the local atrial EGM could possibly be explained by two different mechanism: (1) rounded, low-amplitude slow potentials correlate with the activation of the transitional and/or nodal cells in the context of INE, whereas relatively sharp spiky potentials may represent the insertion of the atrial myocardium into the SP atrio-nodal interface; (2) in the transmural re-entrant circuit, the sharp potential would represent the superficial atrial/transitional cells at the perinodal interface, whereas the low-amplitude slow potentials would correspond to the deeper cells of the INE itself (66–68).

### 5.1 Low-voltage bridge (LVB) strategy

All these findings suggested that the nodal/peri-nodal structures implied in the re-entrant circuit could be visualized during 3D mapping. This is the basal concept of the so-called LVB strategy in mapping and ablation of SP, where the LVB is thought to be the direct visualization of the SP in the area of KT using voltage gradient mapping.

Indeed, after a 3D reconstruction of the endocardial geometry of the right atrium, mapping the voltage of the area of Koch's Triangle, AVN appears as a low-voltage area at the anterior superior tricuspid annulus in the expected anatomic position and the SP associated LVB is observed to connect the high-voltage region at the CS ostium to the AV nodal region. In the first study by Bailin (58), which enrolled mostly adults, 90% of the patients had a discrete LVB that connected the high-voltage region of the coronary sinus ostium to the high-voltage region of the AV node/His. These discrete connections were defined as type I LVB connections. In a minority of patients, the SP-associated LVB had a narrower area and was labelled as type II LVB.

The evidence that the LVB, located within the Koch's Triangle, represented the SP was supported by the following observations: (1) mapping within KT results in a loss of LVB when the SP refractory period is reached, (2) the lack of AVNRT inducibility following successful ablation is correlated with the disappearance of LVB connections, and (3) persistence of an isolated AVN echo beat is associated with incomplete LVB ablation or the presence of a second, separate, and distinct LVB.

The advantage of this novel ablation approach is the ability to visualize the SP within the KT and provide a definitive endpoint for ablation. This can reduce or eliminate the risk of iatrogenic AVB, as the catheter is placed on the LVB rather than on an empirical anatomical location or a location with a characteristic EGM.

Malloy (47) and then Bearl et al. (48) first validated the presence of LVB in children with AVNRT. In these studies, LVB strategy was found to reduce the procedural time, but it needed a learning curve and an “idealization” of the voltage map (increasing high- and low-voltage parameters) in patients for whom a LVB is not immediately obvious. So, in the Bearl's study, the ideal voltage range for each patient stratified by age or weight was reported.

**Figure 3:**
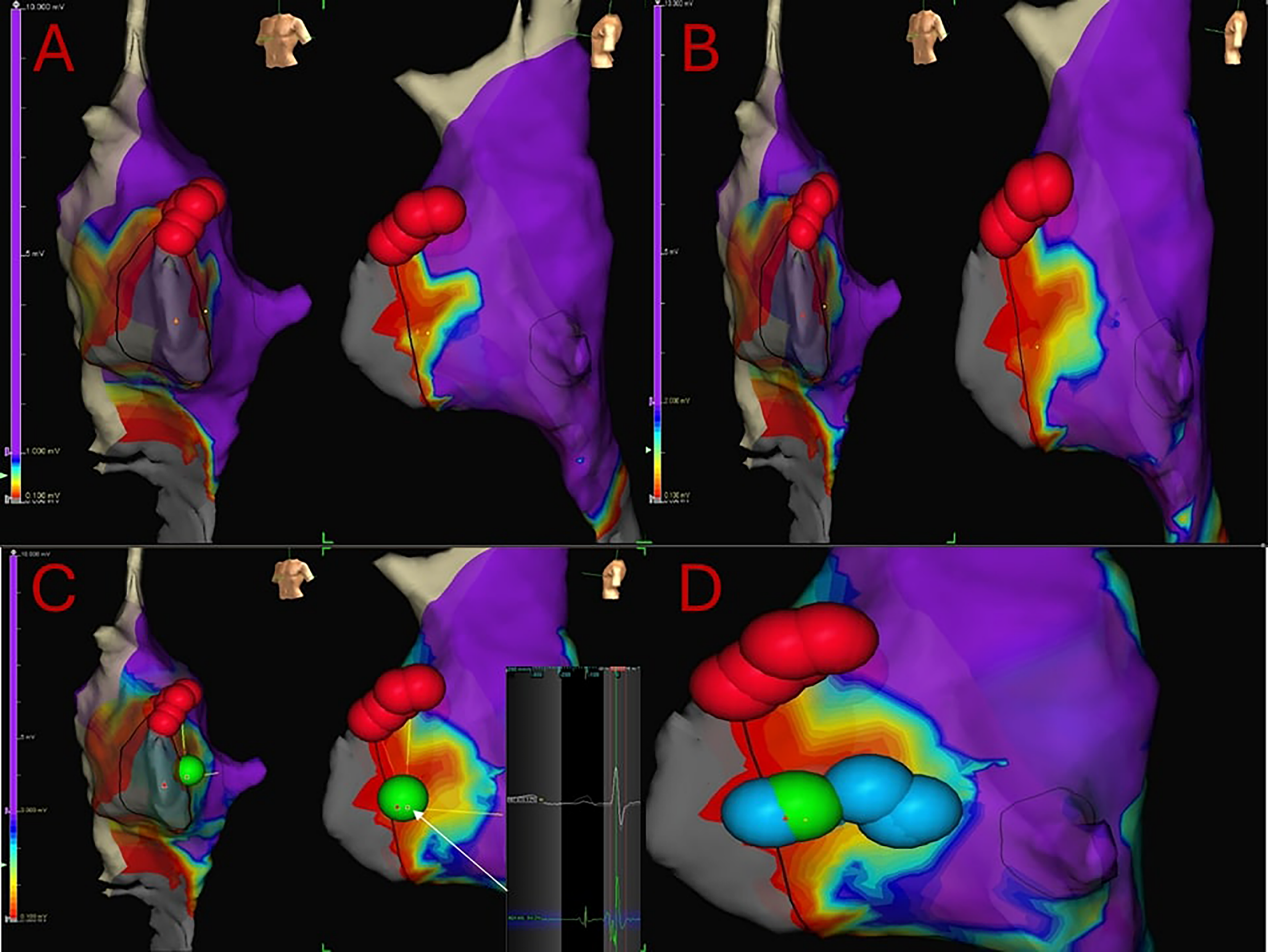
7 years-old, 24 kg girl suffering from both typical and atypical AVNRT refractory to medical therapy. Electrophysiologically guided low-voltage bridge (LVB) strategy, through 3D mapping of the Koch's triangle and the search for the typical “hump and spike” electrograms. Subsequent “linear lesion” cryoablation. **(A)** 3D mapping of the Koch's triangle; voltage bar range of 0.1–1 mV. A type 1 LVB is immediately visible. Red dots: His location. **(B)** Expanding voltage bar range to 0.1–2 mV, an even larger LVB is evident. **(C)** Voltage bar range to 0.1–3 mV. First and successful cryoablation (green dot) delivered in the exact location where the hump and spike electrograms were detected. **(D)** Due to the presence of residual nodal echo beats, a liner lesion cryoablation is the delivered in the Koch's triangle to cover the entire LVB.

Drago et al. later described a 3D mapping strategy using LVB slightly different from the others. Indeed, it takes into account the presence of the so called “hump and spike” potential to confirm the site of SP associated LVB (44, 49, 51) (Figure 3).

In their work, 3D mapping is achieved through the EnSite mapping system™ (Abbott Medical) and usually a deflectable quadripolar (2-5-2 mm) catheter maintained as parallel as possible to the endocardial surface.

After performing the voltage mapping of the right atrium (RA), a very high-density voltage map is created for the interatrial septum (IAS) within the KT. Point collection of at least 130–150 points within the KT is performed, whenever feasible. Interpolation is set at 7 mm, and the interior/exterior projection at 7 mm.

The voltage color bar is then individually adjusted for each patient to highlight a LVB, if present, starting from a voltage range of 0.1–2 mV. Voltage data below the lowest value are shown as grey, whilst those above the upper limit as purple. The values between the two thresholds are displayed as green, yellow, and red. Both high- and low-voltage values are modified until a voltage bridge becomes evident. This adjustment is necessary because of voltage variability in each patient.

Once substrate mapping of the IAS and KT is completed, the width of the voltage color bar (color low and high) is reduced as much as possible (usual range 0.1–1 mV) in order to obtain the most accurate electro-anatomical delimitation of the LVB defined as an area of low voltage, along the base of the KT, that protrudes into the KT connecting high-voltage areas of tissue (Figure 3A). Then, the color bar range (color low and high) is expanded again to highlight those previously identified areas with intermediate voltage, branching off from the LVB area, which could be involved in the slow conduction (Figures 3B,C).

LVBs identified are classified as follows: (1) a type I LVB (a clear, rather large area of low voltage between the CS ostium and the AV node with the base on the edge of the tricuspid annulus) or (2) a type II LVB (a narrow and small area of low voltage between adjacent normal-voltage regions with the base on the edge of the tricuspid annulus). LVB are generally detected with a voltage ranging from 0.12 ± 0.2 to 1.13 ± 0.5 mV.

During voltage mapping the SP potential is searched and its position is analyzed in relation to the area of the LVB. Using this electrophysiologically guided LVB strategy the SP potential is searched to confirm the exact site of LVB in the area of KT.

Using this mapping technique in children with any type of AVNRT, at least one LVB can be found in about 90% of the patients and two LVBs in the others with a higher prevalence of type I LVB in comparison with type II (60 vs. 40%). Interestingly, in atypical AVNRT, Type II seems to be more prevalent than type I, and in children having both types of AVNRT, two LVBs can be found in a higher proportion compared to those with only typical AVNRT. The SP associated double potential can be within the LVB in all children.

In the first study by Drago et al. (49) using this methodology, the new strategy of AVNRT mapping with a combination of 3D mapping (LVB) and electrophysiologically guided mapping (search for JP) ensured 100% mid-term success (1-year FUP).

Results were later confirmed on a large cohort of children (51), with acute procedural success of 99.2% and overall recurrence rate of 2.7%. In this study, type II LVB was found more frequently in children with atypical AVNRT and the presence of two LVBs was found to be much more frequent in patients with both typical and atypical re-entry. In addition, in patients with atypical AVNRT and more than one LVB, there was an increased recurrence rate during mid-term follow-up, suggesting that complex substrates can be more arrhythmogenic. Those results are similar to the findings of a North American multicenter pediatric study, in which the LVB cryoablation strategy was compared with the conventional strategy, showing a similar short-term success rate (LVB 98.5% vs. control 92%) but a considerably lower recurrence rate (LVB 0% vs. control 11%, *P* = 0.006) (46).

However, some authors reported that: (1) the LVB strategy is not so specific to children with AVNRT, and (2) SP potential seems to offer the highest combination of sensitivity and specificity (0.73 and 0.77, respectively) as a marker of AVNRT even in comparison to the others electrophysiological AVNRT characteristics (AH or HA jumps, echo beat, etc.) (69). Actually, these data highlight the validity of the approach by Drago et al., that uses voltage gradient mapping together with the search of the SP potential.

Notably, mapping with a quadripolar deflectable catheter can be “difficult, operator-dependent and time-consuming”. To solve this problem a multi-polar HD multipolar mapping catheter can be used (44, 50). In this way, HD mapping becomes faster, with higher spatial resolution and definition.

HD voltage mapping with HD Grid™ multipolar catheter can the catheter can collect four times the number of points in half the time in comparison with conventional quadripolar 3D mapping. Moreover, in some patients, at the setting of the voltage color bar with which LVBs are clearly visible in the HD mapping, these latter are not visible in conventional mapping. Unfortunately, but rarely, HD mapping can be somewhat traumatic and children, especially the small ones, can experience transient iatrogenic block (44).

Regarding small children (<25 Kgs) (43, 63), LVB, always associated with the peculiar SP EGM, can be clearly visualized using the same voltage range as that one used in older pediatric population and appeared more defined probably due to the absence of acquired low voltage areas in these very young patients. Especially in this population of patients, after the voltage mapping of KT, if the voltage color bar is completely opened, it can be visualized a definite LVB coming from the area of CS ostium to the area of AVN, in which it is possible to appreciate the JP in the proximal part and the HP in the even more proximal part very close the area of AVN. Then, reducing the voltage range as much as possible to obtain the most accurate electro-anatomical delimitation, it is possible to see that the LVB, as defined above, represents the proximal zone where the JP is present.

### 5.2 Sinus rhythm propagation mapping

**Figure 4:**
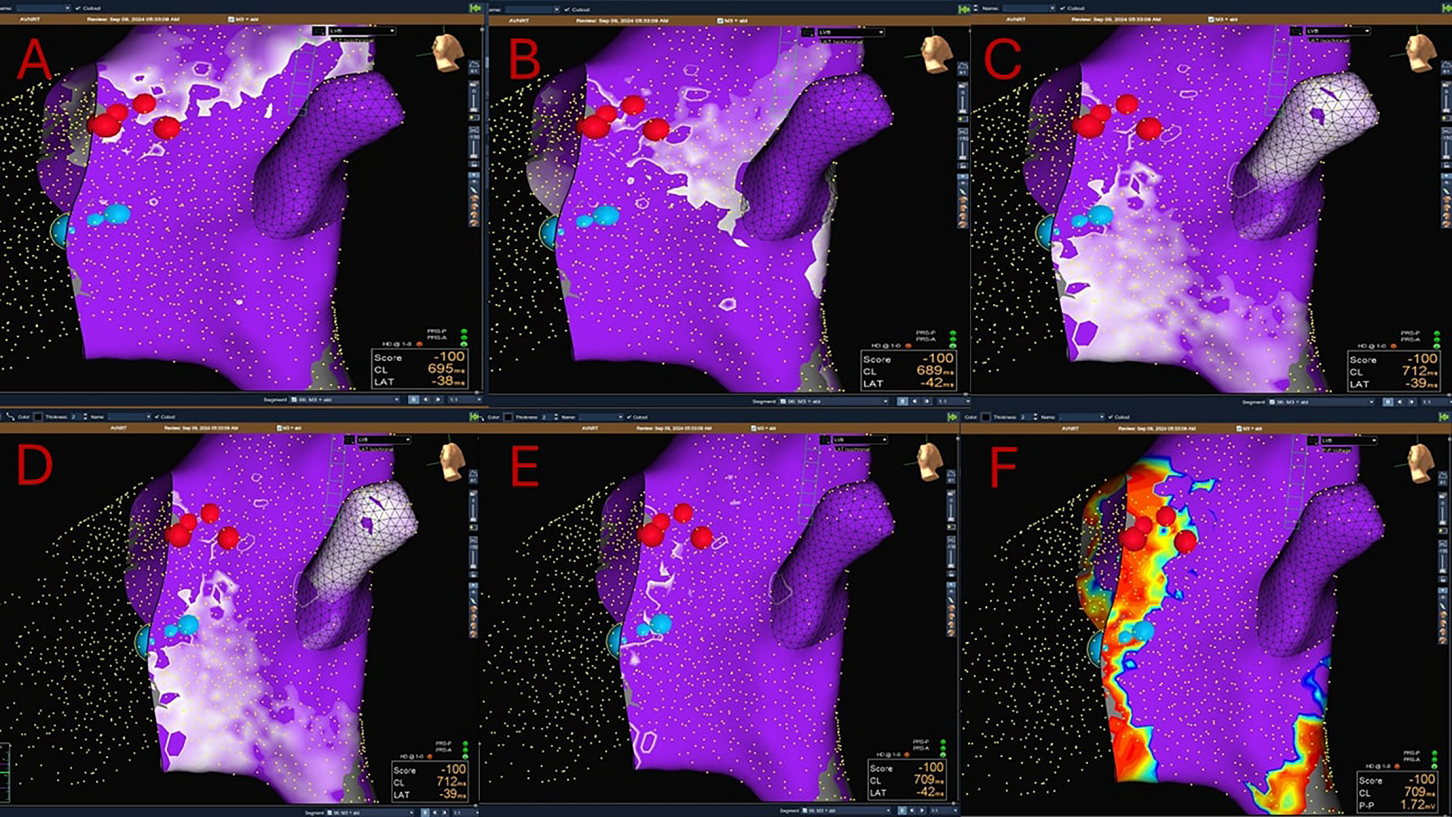
A combined Sinus rhythm propagation mapping and low-voltage bridge (LVB) strategy to guide cryoablation in AVNRT (red dots: His location; blue dots: cryolesions). The wavefront travelling in the inferior–superior direction (via slow pathway) is seen pivoting in the mid-posterior Koch's triangle and colliding with the wavefront moving in the superior- inferior direction (via fast pathway). In the area of collision, a type 2 LVB is identified. Successful cryoablation is performed in this site.

Another described strategy for 3D mapping of the SP is the so-called Sinus Rhythm Propagation Mapping with the identification of Wave Collision or Pivot Point (Figure 4).

Sinus propagation is performed by creating a map of the atrial impulse in the sinus rhythm. Maps are created using a steerable diagnostic catheter with focused data collection within the KT and excluding from analysis ectopic beats. All 3D mapping systems (i.e., EnSite mapping system™ by Abbott Medical, CARTO™ 3 by Biosense Webster Inc, Rhythmia HDx™ by Boston Scientific) can be used (52, 53, 61).

Sinus impulse in AVNRT patients typically splits in two wavefronts that reach separately the area of the KT. One wavefront moves superiorly around the ostium of the SVC and the other along the lateral wall toward the crista terminalis. Then, the two wave fronts enter in the KT moving in opposite directions: one, entering KT from the superior–inferior direction (via the fast pathway), proceeds anterogradely by activating the His bundle (Figure 4A) and retrogradely (superior–inferior direction), colliding with the wavefront coming from the inferior–superior direction (via the SP) (Figures 4B–D).

The area of collision correlates with the location of the SP (52, 53) (Figure 4E). The reason of this is still unclear and it is most likely a surrogate marker.

The *wave collision point* occurs in the mid or posterior-septal portion of the KT, where a SP potential is recorded in most patients. In addition, a marked conduction delay and fractionated, low amplitude potentials are present in this area (52–70).

The presence of *pivot points* is usually found in electroanatomic activation maps of the KT (displaying activation vectors superimposed on standard isochronal local activation maps). It is defined as a marked change in activation direction, in the area of presumed collision of the FP and the SP. Remarkably, after pivoting, the vectors move back to the AV node (55, 70).

Fogarty et al. (53) with the wave collision mapping strategy reported a reduction in the median number of ablations needed to achieve SP modification, but no significant difference in the total number of ablations and longer total procedural times.

Since the LVB and the Sinus Rhythm Propagation Mapping techniques are not mutually exclusive, some authors reported their combined use for AVNRT mapping (54, 55) (Figure 4).

In a study by Van Aartsen et al. (52), the relationship of the propagation maps to both the voltage maps and the successful site of ablation in patients who underwent ablation for AVNRT were retrospectively evaluated. They found low-voltage areas and a wave collision in all patients. The successful ablation lesion was located over a LVB, usually within 4 mm of the wave collision within the KT and superior to it.

Tseng et al. (54) showed comparable procedural times and 100% efficacy both for the study group (propagation map and voltage map) and the control group (anatomic-based approach); however, the number of the cryoapplications and the recurrence rate were lower in the 3D mapping group, suggesting a more precise procedure. Moreover, this strategy was particularly advantageous in patients with multiple SPs. Indeed, in this study if multiple collision points were present, all were marked, and progressively targeted from the furthest to the closest to the His region. Lastly, they also reported that in approximately 30% of patients no LVB was found or the LVB did not correspond to the final successful ablation site.

### 5.3 Other strategies

The omnipolar mapping technology, a new mapping tool available in EnSite™ X EP system (Abbott Medical) can be also used to better localize the SP in patients with AVNRT (55). Omnipolar EGMs are calculated at cliques of 3 electrodes (3 unipoles, 2 orthogonal bipoles) on a multipolar HD-grid catheter, simultaneously analyzing single depolarizations from multiple directions and providing activation direction and voltage. However, omnipolar voltage maps by definition generate the highest EGM amplitude independent of wavefront direction; therefore, it can be less accurate in defining LVBs because low voltage zones in the KT in AVNRT patients are theorized to represent nonuniform anisotropic conduction or collision resulting in atrial EGM fractionation and diminution.

In detail, this technique enables to obtain a more accurate activation vector pivot (pivot point), defined as a ≥45-degree change in activation direction within the KT. Moreover, it allows the use of an algorithm that distinguishes near-field EGMs (high-frequency) from far-fields (low-frequency). Indeed, the “peak frequency analysis”, which is an automated identification of high frequency EGMs, can be used create a voltage map restricted to a peak frequency >340 Hz, which in turn can identify precisely the successful ablation site for AVNRT. Probably, it represents the atrial or transitional cells' EGMs connecting the inferior input to the SP and not the true SP (right inferior nodal extension) (55, 71). However, the target of ablation of the SP can also be the site that displays a relatively lower peak frequencies, within the range of 180-210 Hz, that actually is a pseudo far field potential, related to the presence of cells with slow upstroke of action potential and low conduction velocity, typical of the cells of the right inferior extension of the AV node (SP) (72).

O’Leary et al. (45) used both the bipolar and the omnipolar mapping technology and found that, omnipolar activation vector pivot (pivot point) had the highest positive (81%) and negative predictive values (100%) for identifying AVNRT cases and had a median distance of 1 mm from the effective ablation site. The authors also showed that conduction velocity within the TK was significantly slower in patients with AVNRT than those without AVNRT, but it was not useful in identifying the successful ablation site, leading to the conclusion that globally reduced KT conduction velocity may represent a marker of AVNRT substrate without necessarily identify precise ablation sites. Moreover, they found that the bipolar voltage restricted to a peak frequency >340 Hz had an optimal discriminatory performance to identify successful ablation sites.

Another proposed strategy for AVNRT mapping relies on the Ripple mapping software (CARTO™ 3, Biosense Webster Inc) (56, 73). It is obtained with a multipolar mapping catheter, used to collect points along the right septum during sinus rhythm or during atrial pacing to increase the density of atrial activation. Atrial signals with voltages greater than 0.06–0.08 mV are utilized for the creation of the map. This software displays each EGM at its 3D coordinate as a bar changing in length according to its voltage–time relationship. This allows prolonged, low-amplitude signals to be displayed entirely, helping recognize propagation in low-voltage areas. A benefit of this technology is that manual annotation or interpolation are not needed. Howard et al. (56) compared this strategy to the standard electro-anatomical mapping in children and found that it helped to better localize the exact location of the SP (successful ablation lesion in ≈90% of cases within 4 mm of the predicted site, reduced variability in number of test lesions until success).

### 5.4 Fluoroscopy minimization with new mapping systems

As mentioned above, the new available technologies in mapping systems play a key role not only in guiding precise substrate ablation but also in minimizing/eliminating fluoroscopy, which is of particular importance in children.

In a study by Reddy et al., authors demonstrated a reduction in fluoroscopy use in the 3D mapping group, along with a slight improvement in acute success rate and a significant reduction of recurrences (46).

Similarly, in the studies by Drago et al., zero fluoroscopy or near-zero fluoroscopy was achieved with the use of a 3D mapping strategy (43, 44).

Moreover, in another single-center study by Topalović et al., zero-fluoroscopy ablation of AVNRT in the pediatric population was found a feasible, effective and safe treatment option, with a 100% acute success rate and a 98.7% chronic success rate (74).

Cui et al., using a 3D mapping system demonstrated that fluoroscopy exposure in children can be completely avoided without affecting the safety and efficacy of RF ablation (75).

Lastly, in a recent meta-analysis on zero/minimal fluoroscopic approaches during SVT ablation in both children and adults, it was demonstrated that this approach reduces radiation exposure and ablation time without compromising the acute and long-term success or complication rates (76).

### 5.5 Role of intracardiac echocardiography (ICE)

In challenging cases, the use of ICE has been found to improve outcomes of AVNRT ablation, since it provides real-time visualization of intracardiac structures and the ablation catheter. Indeed, data in adult patients proved that it can reduce mapping and ablation time, radiation exposure, and RF deliveries (77, 78).

Although widely used in adult EPS, ICE utilization in pediatric patients raised concerns for complications such as valvular and vascular injuries. On the other hand, when dealing with complex anatomic structures, such as in repaired CHD patients, ICE may be a valuable tool to directly visualize cardiac suture lines, baffles, and conduits. In this setting, it can be used to guide trans-baffle and trans-conduit puncture and provide precise localization of remote arrhythmic substrates. Moreover, it helps to monitor lesion formation and facilitate rapid diagnosis of complications (79).

In a recent study by Headrick et al. including 335 pediatric and CHD ablations, the use of ICE was safe and reduced procedure duration, fluoroscopy exposure, and arrhythmia recurrence compared to no-ICE control group; in detail, in AVNRT cases, it significantly reduced procedure duration without negatively affecting success rates (80).

## 6 Transcatheter ablation

In recent years, transcatheter ablation of AVNRT has become the treatment of choice in large enough children (i.e., >15 kg) and in patients with CHD; indeed, as discussed, technological improvements have made this procedure highly safe and successful. In recent guidelines catheter ablation is recommended for symptomatic, recurrent AVNRT patients. Chronic medical therapy, either with non-dihydropyridine calcium channel blockers (in patients without left ventricular dysfunction) or with beta-blockers, should be considered when ablation is not desirable or feasible (7, 81). As regards smaller children, catheter ablation is rarely used and is recommended when medical therapy is either not effective or associated with intolerable adverse effects (7).

Radiofrequency (RF) ablation was first used in the 1990s, while cryoablation was introduced in 2003 (82–84) and nowadays represents the most used energy for AVNRT ablation in pediatric patients (2, 44, 46, 49, 85). Indeed, its specific features, such as reversible cryomapping, cryoadhesion and the precision in lesion delivery, made this technique very appealing to decrease complications and fluoroscopy time in pediatric patients (86).

### 6.1 Cryoablation

Cryoablation is usually performed using a steerable cryoablation catheter (Freezor™, Medtronic Cryocath LP, Montreal, Quebec, Canada, 4–8 mm tip size catheters). As regards tip sizes, 4 mm tip catheters are usually chosen for patients weighing <30 kg or with very small KT dimensions.

As mentioned, the use of cryoenergy allows the use of cryomapping, and different types of mapping have been described.

“Fixed cryomapping” is performed by reducing the tip temperature to −30°C for a maximum of 60 s; “step-by-step cryomapping” is performed by progressively decreasing the tip temperature by 10°C every 5 s from −30 to −70°C.

During cryomapping, repeat extrastimulus testing or ramps can be performed using the diagnostic catheter placed in the high right atrium to assess tachycardia inducibility or the absence of AV nodal SP conduction. If cryomapping is effective (i.e., no AH jump, no re-entry beats, and no inducibility of tachyarrhythmia), the tip temperature can be further reduced to create a permanent lesion. In contrast, if cryomapping produces unwanted effects (e.g., transient high-degree AV block, lengthening of the PR interval), cryoapplication can be discontinued to allow tissue rewarming and reversibility of electrical function loss. Subsequently, the cryocatheter is repositioned to another target site, and cryomapping repeated. As a result, permanent AV node damage has never been reported with this energy source.

Several techniques have been described for cryoablation as well.

**Figure 5:**
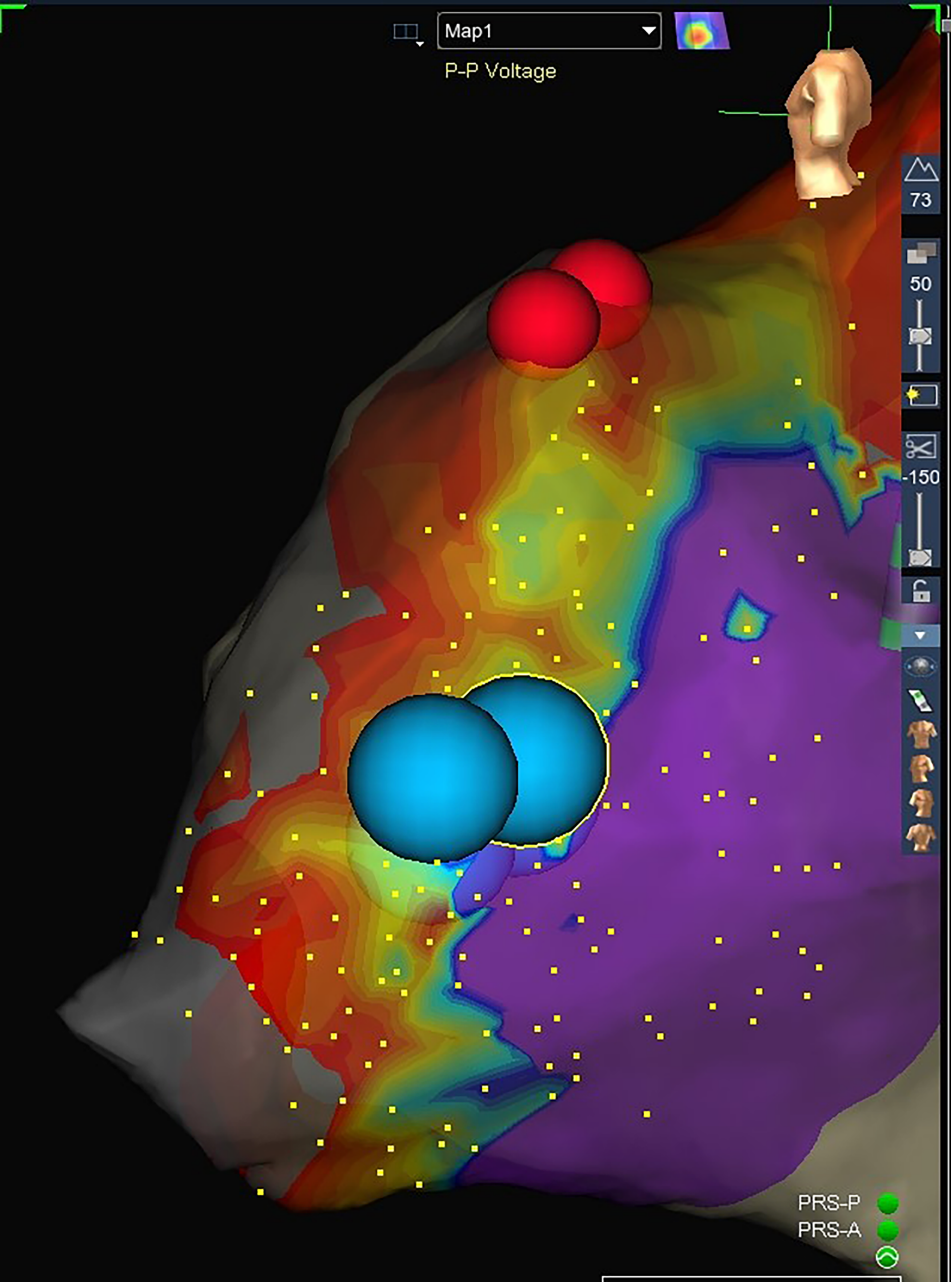
Single cryoablation followed by cryobonus.

The focal lesion technique consists of delivering a single cryoablation at −75°C–80°C for 4–8 min followed by one or more cryoenergy applications (cryobonus) in the same spot to consolidate and slightly enlarge the cryolesion (Figure 5). Importantly, after each delivered cryoablation, repeated extrastimulus testing or ramps may be conducted to reassess tachycardia inducibility or the absence of SP conduction.

In this regard, Drago et al. demonstrated that 8-min cryoapplications together with the use of cryobonuses improve the chronic success rate, maintaining an excellent safety profile (63).

Alternatively, the extended focal lesion strategy relies on a single lesion followed by one or more cryoenergy applications delivered slightly anterior and posterior to the first successful or apparently successful lesion.

Another option is the linear lesion technique (or 3D-guided “high-density linear lesion” [HDLL) (Figure 2D). It is carried out delivering multiple overlapping cryolesions from the ventricular to the atrial side of the tricuspid annulus, with a final cryolesion at least 10 mm long, obtaining an overlapping of 3D spheres/lesions of at least half of their diameter or at least 2 mm.

The extended HDLL is produced when extending the lesion anteriorly and posteriorly to the original HDLL to achieve successful cryoablation of the arrhythmia (49, 87–92).

In a study by Czosek et al., linear lesion cryoablation improved procedural outcomes and recurrence rates compared to single-lesion methods (92).

It has also been reported that cryoablation with a 6-mm tip catheter provides higher acute and chronic success rates than 4-mm tip catheters, and 8-mm tip catheters may further reduce the risk of recurrence in children with AVNRT, while maintaining an excellent safety profile. However, with the widely used Medtronic cryocatheters, the 8-mm tip does not provide the cryomapping feature (93, 94).

Also, double and triple freeze-thaw cycles (FTC) have been reported to diminish recurrences in AVNRT patients, probably because of the larger lesion volumes achieved. However, issues regarding transient AV block and possible coronary artery injuries have been raised (95, 96).

From its introduction in 2003, the use of cryoenergy for AVNRT ablation increased and, with growing experience using this technique, outcomes have improved.

Indeed, in the early era, when 3D mapping systems were not widely available and used, cryoablation of SP substrates was reported to carry higher recurrence rates than RF (87, 89, 97, 98).

However, in the following years several studies regarding cryoenergy use for AVNRT ablation showed acute success rates of >95% and comparable recurrence rates to RF, even though with an increased variability among the studies (8% recurrence rate, range 0%–23%), probably related to different catheter tips and protocols (number, duration, and extension of cryolesions) (47, 49, 92–94, 99–101).

Finally, in the era of 3D mapping systems, cryoablation of AVNRT substrates has been reported to produce chronic success rates >95% (Table 1).

For example, in the multicentric MAP-IT registry (2), cryoenergy was most commonly used for AVNRT ablation (53% of cases) and no differences in acute success as compared to RF were reported (acute success 99% for both energy sources; success at FUP 95% for cryo and 92% for RF).

In the EUROPA registry (1), even though cryoenergy was used in a minority of patients, its procedural success and tachycardia recurrence were comparable to RF. Therefore, it was suggested as a possible primary energy source for the ablation of tachycardia substrates near the cardiac conduction system, such as AVNRT.

Regarding monocentric studies, Drago et al. showed their growing experience and improved results with cryoablation, reaching chronic success rates at mid/long-term FUP ≈100% (43, 44, 49, 51, 88–91).

Similarly, two large monocentric studies (46, 85) demonstrated that the combined use of cryoenergy and non-fluoroscopic 3D mapping systems ensured acute success rates of 100% and recurrences ranging from 0% to 4.4%.

### 6.2 RF ablation

RF energy is another available option of trans-catheter ablation of AVNRT. It is delivered and guided by monitoring the temperature at the catheter tip, which is usually limited to a maximum of 55°C–60°C with a preset maximal power of <55 Watts (usually 20–40 W according to patients’ body weight and operators’ choice) (97, 102–104).

When RF is applied in the area of the SP, an accelerated junctional rhythm (<120 bpm) typically occurs, and RF energy is stopped if VA prolongation/block with junctional rhythm occurs. In addition, the RF current is terminated immediately in the event of an increase in impedance, displacement of the ablation catheter, or prolongation of the PR interval. If junctional beats are not evoked, RF delivery is usually interrupted, and the catheter is repositioned. Otherwise, RF deliveries are applied from 30 s to 60 s (105, 106).

RF is mostly delivered through non-irrigated-tip catheters; however, in the recent MAP-IT trial, irrigated tip RF catheters were used in 25%–30% of patients, with a 99% success rate and no reported complications (2).

In the early era of catheter ablation in children, the main multicenter studies focused only on the outcomes of RF ablation. In particular, the Pediatric Radiofrequency Catheter Ablation Registry (PCAR) (107) reported a success rate for AVNRT ablation of 95%–99%, with an improvement across the years, and a complication rate of 3%–5%. Similarly, in the Prospective Assessment after Pediatric Cardiac Ablation (PAPCA) (108), the registry success rate was 97% and the complication rate 2.9%–4.2%, with 2.15% for AV block.

In the PAPCA registry, the recurrence rate was 4.8% at 1-year FUP (108). In concomitant monocentric studies, results were comparable: acute success rate slightly above 95% and recurrence rate of 5%–10%, with a non-trivial occurrence of AV block (98, 109).

In recent years, a large Chinese registry concerning RF use in the treatment of arrhythmias in pediatric patients (110) showed that the acute and long-term success rates of AVNRT ablation were 99.3% and 95.0%, respectively. However, AVNRT was the most likely substrate to have serious complications, which occurred in 29 patients (0.6%), with 7 complete AV blocks (6 of them requiring permanent pacemaker implantation).

A remote magnetic navigation system to deliver RF has also been described as an ablation tool in children with AVNRT. The remote magnetic navigation ablation catheter has a flexible, magnetically enabled distal tip; therefore, a magnetic force can be used to navigate toward and hold the catheter with high stability in the ablation area (111, 112). This strategy had already demonstrated improved lesion formation and superior safety compared to manual RF ablation; moreover, in a study by Noten et al. (113), it was compared to other catheter ablation strategies and proved to reduce recurrences compared to both manual-guided RF ablation and cryoablation.

It must be highlighted that, regardless of the type of energy used, some issues remain.

First, recurrences remain not negligible and a relevant part of them can occur very late, >5 years post ablation (100, 114). In this regard, many have tried to predict the risk of recurrence; however, neither the presence of jump nor a single echo beat after catheter ablation have demonstrated to be reliable predictors (26, 115). However, more extensive data on very long-term follow-ups is lacking.

Secondly, some authors pointed out that over a very long-term follow-up, the risk of late pacemaker implantation after AVNRT ablation is low but significantly higher than that in the general population. In addition, this risk is even higher than the risk of acute, periprocedural pacemaker implantation (114, 116). However, consistent data on this topic is still lacking.

All considered, a reasonable option for children is to perform SP modulation instead of complete SP ablation, especially when multiple ablation lesions are needed, and RF is used. In these particular cases, the presence of single nodal echo beats in the absence of sustained SP conduction is an acceptable endpoint (7, 117).

## 7 Special considerations in CHD

AVNRT may develop in patients with different types of CHD at any age and represents the third most common form of arrhythmia in children with CHD (after atrioventricular reentry tachycardia and macro-reentrant atrial tachycardia) (23).

AVNRT ablation in these patients is even more valuable than in normal hearts, since the tachycardia may be detrimental to already fragile hemodynamic status and antiarrhythmic drugs have limited efficacy and more side effects than in patients with structurally normal hearts. However, catheter ablation may be challenging, and experience remains limited (22, 118).

In CHD patients, special considerations relate to the localization of the specialized conduction system, which may be atypical and less predictable or less accessible. The former problem occurs more frequently in patients with AV septal defects, CC-TGA, heterotaxy syndromes, tricuspid atresia and double inlet ventricles and implies lower success rates and higher risks of AV block during ablation. The latter mainly occurs in D-TGA patients after Mustard or Senning operation and in patients with Fontan circulation. Indeed, accessing to the conventional SP region is complex in these cases as it most often requires a trans-baffle puncture for a direct antegrade approach. Moreover, in Mustard/Senning operation, SP ablation occasionally may require additional ablations to its ‘left-sided’ atrio-nodal input from the systemic venous aspect (24, 119, 120).

In general, ablation outcomes in CHD patients are poorer than in normal hearts, with acute success rates around 80% and major complications in 4%–5% (119, 121).

In addition, as demonstrated by Papagiannis et al, the complexity of the CHD influences electrophysiological characteristics and ablation results. Indeed, patients affected by complex CHD have more frequently atypical AVNRT and are usually ablated at younger ages. Furthermore, ablation success rate is significantly lower (82% vs. 97% in simple CHD), recurrences higher (18% vs. 10%) as well as complications, with AV block requiring pacing in 10% (only with RF ablation) vs. 0% in simple CHD (22).

As regards specific CHD: CC-TGA is usually characterized by the abnormal location of AV conduction tissue and a marked fragilily of it, which warrants a supreme care to avoid AV block and obviate the need for a permanent pacemaker. The compact node generally occupies a more anterior location, outside of the KT. In CC-TGA with situs inversus (I,D,D) ablation is usually performed in the left side, while in situs solitus (S,L,L) in the right side; for both, usually in the mid/posterior septum, even though cases of ablation in the anterior septum or even in a superior location medial to the right atrial appendage have been described (22, 24).

In D-TGA after Mustard or Senning operation the intra-atrial baffle prevents direct entrance to the KT from a femoral venous approach. Therefore, either a retrograde trans-aortic approach to the pulmonary venous side of the baffle or a trans-baffle approach are required (22, 122).

In patients with AV canal defects, the connecting AV node is displaced to an inferior location in the KT or even inferiorly to the KT, between the atrial sinus septum and the inlet of the muscular ventricular septum. The non-branching and branching bundles are typically exposed by the defect, i.e., not covered by valvular tissue, making them more susceptible to damage. Therefore, RF is not recommended by most authors, while the use of cryoenergy has been described more extensively (22, 24, 119, 123).

In patients with Single Ventricle Physiology (included those after Fontan operation) the reported outcomes are the worst, because of the uncertainty of the nodal/perinodal tissue and the lack of conventional landmarks of the KT, especially in heterotaxy and unbalanced AV canal defects, in which there is no KT to assist with AV node localization (22, 24). Furthermore, in dextrocardia or heterotaxy syndrome, the AV node may also be left-sided and rarely duplicated. Also, a conventional femoral approach may be inadequate due to venous anomalies (inferior vena cava absence in left atrial isomerism) (124). In addition, in patients with Fontan circulation, a trans-baffle puncture is required and carries the risks of potentially catastrophic consequences, such as intrathoracic hemorrhage (24, 119, 124).

In patients with tricuspid atresia, successful SP ablation may be sometimes achieved from the left side of the atrial septum, thus through a trans-septal or retrograde approach.

In Ebstein anomaly right atrial dilation and tricuspid valve displacement can distort the KT anatomy, but usually the sites of successful SP ablation are conventional (24).

## 8 Conclusion

AVNRT is a common SVT in children and its invasive management has deeply evolved in recent years. Mapping strategies in pediatric patients have significantly improved, leading to a more precise localization of the ablation substrate, with minimal use of fluoroscopy. It enabled us to perform fewer ablation lesions, reducing complications, and to avoid unnecessary radiologic exposure in growing human beings. Catheter ablation itself has now become extremely safe and effective, with reduced AV block rates after the spread of cryoenergy. However, overall risk of AV block and long-term consequences of catheter ablation in the pediatric age remain open issues. Moreover, in CHD patients ablation remains challenging and experience limited. Therefore, even with improved technologies and expertise, caution should be exercised in children, and a risk-benefit evaluation should be considered.
